# Improving Outcomes Defending Patient Safety: The Learning Journey in Robotic Liver Resections

**DOI:** 10.1155/2019/1835085

**Published:** 2019-04-08

**Authors:** Paolo Magistri, Gian Piero Guerrini, Roberto Ballarin, Giacomo Assirati, Giuseppe Tarantino, Fabrizio Di Benedetto

**Affiliations:** Hepato-Pancreato-Biliary Surgery and Liver Transplantation Unit, University of Modena and Reggio Emilia, 41124 Modena (MO), Italy

## Abstract

**Background:**

While laparoscopy is currently adopted for hepatic resections, robotic approaches to the liver have not gained wide acceptance. We decided to analyze the learning curve in the field of robotic liver surgery comparing short-term outcomes between the first and the second half of our series.

**Methods:**

We retrospectively reviewed demographics and clinical data of patients who underwent robotic liver resection at our institution from July 2014 through September 2017. 60 patients diagnosed with primary or secondary liver neoplasms or hydatid disease were included in this study. ASA PS >3, heart failure, respiratory insufficiency, and general contraindication to pneumoperitoneum were exclusion criteria.

**Results:**

60 patients were included. We observed a statistically significant decrease in operative time (p<0.001), intraoperative blood loss (p=0.01), and postoperative complications (p<0.001) after 30 cases. From the interpretation of the CUSUM curve, the time of operation appears to be significantly reduced after the first 30 operations.

**Discussion:**

This is the first European analysis of the learning curve for robotic liver resection in an HPB and liver transplant referral center. However, more studies are needed to confirm such results outside a HPB referral center. This is crucial to develop formal credentialing protocols for both junior and senior surgeons.

## 1. Introduction

Over the past decade, the role of laparoscopic liver surgery has been discussed in three international consensus meetings held in Louisville, Morioka, and Seoul [1]. As a result, consensus guidelines were produced in another international meeting held in Southampton in 2017 [2]. It has been demonstrated that laparoscopic surgery is currently the gold-standard for left lateral sectionectomy, and the presence of at least 2 surgeons proficient in laparoscopic liver surgery (LLS) is recommended in each HPB center [2, 3]. Conversely, robotic approaches to the liver have not gained wide acceptance. Experience on the* da Vinci*® platform is growing among many surgeons starting from the pioneering work of Giulianotti and colleagues [4], but the learning curve for proficiency has yet to be defined. According to the report from the second international consensus conference held in Morioka [5] robotic surgery is considered to be IDEAL stage 2a [6] (Development)—especially in regard to instrumentation. In other words, it requires both ongoing institutional ethical approval and a reporting registry of all cases before beginning to perform this procedure. Therefore, it is crucial to define learning and proficiency gain curves to ensure patients' safety while developing this technology. We herein report our experience with robotic surgery of the liver from a high volume and tertiary referral center for hepatobiliary surgery and liver transplantation.

## 2. Methods

### 2.1. Management of Liver Disease at University of Modena and Reggio Emilia

The hepato-pancreato-biliary robotic program started at University of Modena and Reggio Emilia on July 2014 and 136 procedures have been completed so far. Indication to surgical resection in our center is always discussed in a multidisciplinary meeting involving surgeons, radiologists, hepatologists, and oncologists, as already described [7]. All the robotic surgical procedures were performed by the same surgeon (FDB), who completed robotic training for the da Vinci Si platform ® (Intuitive Surgical Inc., Milford, CT). He is a fully trained surgeon in HPB and liver transplant, with previous experience in minimally invasive surgery of the liver. All procedures were approved by the institution's supervisory committee, and this study was approved by the institutional review board.

All patients diagnosed with either HCC, cholangiocarcinoma, metastases, adenoma of the liver, or hydatid disease were evaluated by preoperative examinations to determine the liver function with conventional liver function tests (including Child-Pugh classification), serum alpha-fetoprotein (AFP), hepatitis B surface antigen, and anti-hepatitis C virus antibody measurements.

Portal hypertension was assessed by platelet count, gastro-duodenal endoscopy (EGDS), and measurement of hepatic vein portal gradient (HVPG) when needed. Moreover, selection criteria included compensated cirrhosis (both Child A and Child B patients) and non-cirrhotic liver disease, esophageal varices ≤ grade 1, platelet count ≥ 50 × 10^9^ /L, American Society of Anesthesiologists (ASA) Score ≤3, and HVPG <14 mmHg. To determine the extent of resection all patients underwent triphasic computed tomography (CT) scans, and/or contrast enhanced magnetic resonance (MR) imaging ASA Physical Status (ASA PS) >3, heart failure, respiratory insufficiency, and general contraindication to pneumoperitoneum were exclusion criteria for minimally invasive liver surgery (MILS) [7]. Patients affected by tumors showing extensive sub glissonian infiltration or infiltrating major hepatic vessels were also excluded from MILS.

Each patient signed an informed consent, at least one day before the surgical day, which included the authorization to keep audio-visual material of the surgical procedure and the perioperative and follow-up data in our institutional prospectively maintained database. After surgery, all patients were followed at our outpatient clinic at 3- or 6-month intervals. Follow-up examinations included clinical examination, liver function tests, and imaging according to the primary disease and to the multidisciplinary consensus.

### 2.2. Patient Selection and Data Retrieval

We retrospectively reviewed demographics and clinical data of patients who underwent robotic liver resection (RLR) at our institution from the start of our robotic program in July 2014 through September 2017. Fenestrations of simple or complicated hepatic cysts were excluded from this study. We retrieved demographics and data from past medical history from patients' medical files, while intraoperative data were prospectively collected. Estimated blood loss was calculated as the difference between volume in the suction system and irrigation volume. Operative time was considered from the induction of the pneumoperitoneum to the suture of the trocar insertion sites, thus including the docking time. Postoperative complications were classified using the Clavien-Dindo Classification for Surgical Complications [8].

Prospectively collected data, including intraoperative variables, postoperative complications, and pathological findings, were analyzed retrospectively.

### 2.3. Surgical Technique

RLR procedures were performed using the daVinci Si Surgical System. Patients are usually positioned in supine 20° to 30° anti-Trendelemburg and can be slightly rotated to the left to allow an easier access to right and posterior segments. The pneumoperitoneum is induced with the Verres needle technique, from the left upper abdominal quadrant (Palmer's point), in patients not presenting with splenomegaly or suspect for abdominal adherences from previous surgeries. An open approach to induce the pneumoperitoneum is preferred in those cases (Hasson's technique). Constant endoabdominal pressure is kept with the use of the automated insufflator AirSeal™ (Surgiquest). Exploratory laparoscopy is always performed before docking the patient cart of the robot. The disposition of the trocars varies according to patient peculiar conformation and lesion localization. Intraoperative ultrasound (US) is always performed to better define tumor size and position, and to assess the correct transection plane, as already reported [9], thanks to the image-fusion between the scope and the US. Parenchymal transection is performed with a combination of monopolar and bipolar energy, and with the use of daVinci Harmonic ACE™ (Ethicon, Somerville, NJ) for deeper layers. Although the robotic platform still does not support liver-specific articulated devices for parenchyma dissection like the Cavitron Ultrasonic Surgical Aspirator (CUSA), the correct use of the available tools allows a safe dissection. For example, the Maryland forceps can be used as a right angle to precisely dissect small vessels in the parenchyma thanks to the 7 degrees of freedom of the robotic instruments. After the sample is extracted, hemostasis and biliostasis are perfected with fibrin glue and a JP drain tube is usually left in place.

### 2.4. Statistical Analysis

Continuous variables are expressed as mean ± standard deviation (SD) or median and range and compared using Student's t-test. Categorical variables were compared using the chi-square test with Yates's correction as appropriate. Statistical significance was set for p<0.05. Statistical analysis was performed using SPSS Statistics version 19.0 (IBM, Armonk, New York, USA).

Cumulative Sum Control Chart (CUSUM) analysis was proposed by Page in 1954 for monitoring small shifts in mean in any production process.

We applied the CUSUM analysis in monitoring performance of our surgical procedures in order to detect adverse events and trends of unacceptable outcomes. The mean of strategic variables was calculated and plotted against the number of procedures performed.

At acceptable level of performance, the CUSUM curve is flat, while at unacceptable levels of performance the curve slopes upward or downward and eventually crosses a decision interval. While this occurs, the CUSUM chart indicates unsatisfactory performance.

## 3. Results

### 3.1. Demographics

67 patients underwent robotic liver surgery in the study period. After the exclusion of simple and complicated cyst fenestration, 60 patients were included in this study and divided into two consecutive groups, the first and the second 30 procedures, to investigate the effect of a mid-term learning curve (Table 1) [10]. Two patients were scheduled for robotic surgery in the first period but the procedures were then aborted due to peritoneal metastasis at the exploratory laparoscopy. No statistically significant difference can be identified in terms of age, gender, degree of cirrhosis, surgical procedure, and preoperative comorbidities. Among the subgroup of bi-segmentectomies, we found that left lateral sectionectomies were performed more frequently in the first period in a statistically significant fashion (p=0.01). The final number of resections in the first period was 31, since one patient received two wedge resections in the same procedure, while no multiple resections were performed in the second period.

### 3.2. Intraoperative Outcomes

A statistically significant improvement was found comparing estimated blood loss and operative time between the two periods. In detail, we observed a difference of 118.56 minutes in the operative time between the first and the second 30 robotic cases (p<0.001). Similarly, estimated blood loss decreased from 484.3 mean mL to 337.3 mean mL (p=0.01).

### 3.3. Postoperative Outcomes

There was no difference in terms of postoperative in-hospital stay, as shown in Table 2 [4.9 days (range 2-13) and 4.9 days (range 2-12) in the first and second period, respectively]. Conversely, a slight but statistically significant difference was demonstrated in the incidence of low grade postoperative morbidity, classified as Clavien-Dindo classes 1 and 2. High grade postoperative complications (Clavien-Dindo classes 3 and 4) and readmissions were uncommon and did not differ between the two periods. Final pathology confirmed that the two populations were homogeneous in terms of indications to surgery.

### 3.4. Difficulty Index Analysis

**Figure 1:**
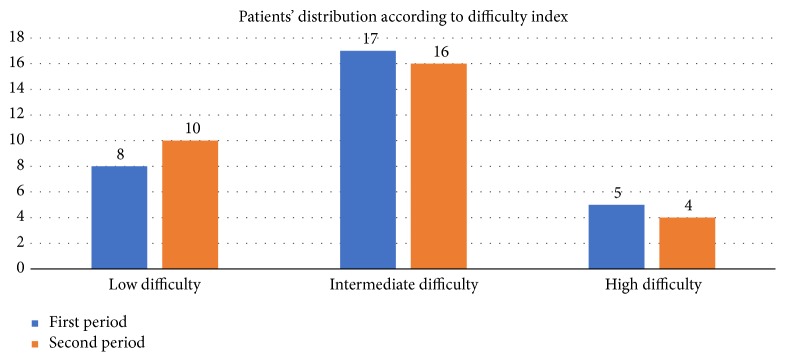
Distribution of patients according to the difficulty index.

We retrospectively analyzed the difficulty index of the cases included in this study according to the scoring system developed and further validated for laparoscopic liver surgery by Wakabayashi and colleagues [11, 12]. The final score is obtained from the sum of 5 difficulty indices: tumor location, extent of hepatic resection, tumor size, proximity to a major vessel, and liver function. The difficulty level is then stratified into 3 categories: low (score 1 to 3), intermediate (score 4 to 6), and high (score 7 to 10). Over the study period 30% of the procedures were staged as low level difficulty, 55% intermediate level of difficulty, and 15% high level of difficulty (see Table 3). We found that low difficulty cases were 8 and 10 among the two groups, respectively (p=0.58), the intermediate cases 17 and 16, respectively (p=0.79), and the high difficulty cases 5 and 4, respectively (p=0.72) (Figure 1, Table 2). The most frequent difficulty score was 5 in both series.

### 3.5. Learning Curve Analysis with the CUSUM Curve

Cumulative Sum Control Chart (CUSUM) was developed for monitoring the mean of following continuous variables: length of hospital stay, operation time, and intraoperative blood loss.

**Figure 2:**
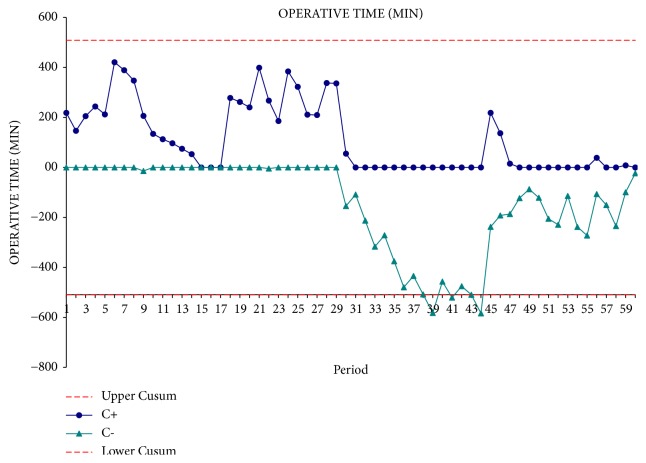
Operative time CUSUM curve.

**Figure 3:**
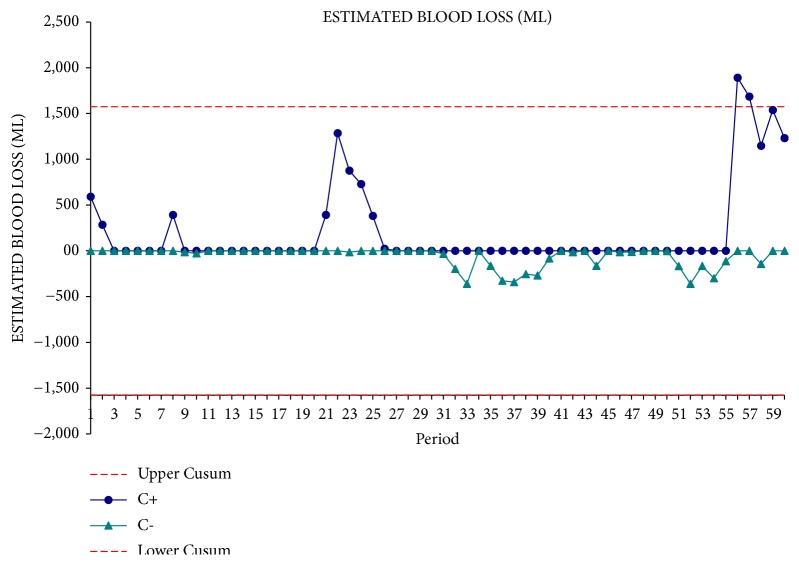
Estimated blood loss CUSUM curve.

**Figure 4:**
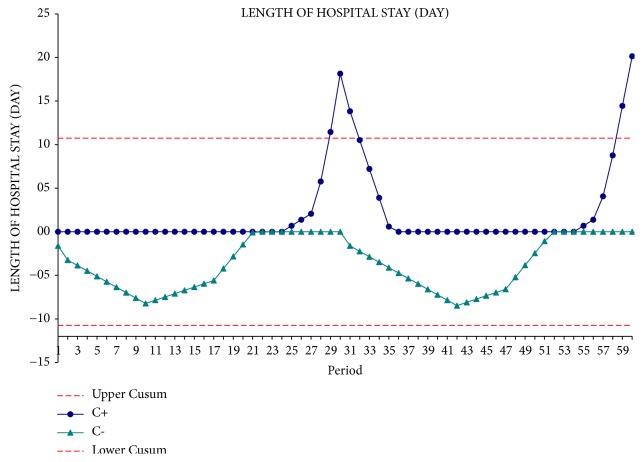
Hospital stay CUSUM curve.

From the interpretation of the CUSUM curve, the time of operation appears to be significantly reduced after the first 30 operations. In the second phase of the experience, operative time reaches a plateau that corresponds to the mean operating time (Figure 2). The blood loss curve remained stable towards the average value that corresponds to 410 ± 393 cc (Figure 3). The curve of the hospital stay followed a trend similar to that of blood loss (Figure 4). This underlines the fact that intraoperative blood loss is a prognostic factor capable of affecting patient's outcomes.

## 4. Discussion

One of the limitations to the diffusion of robotic surgery is the definition of a credentialing training. Secondly, surgical instruments dedicated to minimally invasive liver surgery are still lacking, and no tools comparable to open parenchymal transection devices have been developed for the robotic platform. Moreover, no strong evidences on indications or procedures have been produced. Although some literature has shown comparable outcomes between robotic and laparoscopic hepatic surgery, such results may not be generalizable [13, 14]. It has been claimed that financial burden may be a limitation to the diffusion of robotic surgery [15]. However, the lower rates of postoperative morbidity after robotic resection, including conversion to open, demonstrate that the initial cost of purchasing the robot can be overcome by faster return to daily activity compared to the open approach group (22% versus 40%; p=0.047) [16, 17]. In this series we had to convert the resection from robotic approach to open in one case of right posterior sectionectomy due to gas embolism. The source of the embolism was probably an intra-parenchymal hepatic vein rupture while applying a clip. The patient was desaturing although the bleeding was immediately controlled; therefore we agreed with the anesthesiologist to convert to open and quickly complete the resection. The postoperative course was uneventful and the patient was ultimately discharged on postoperative day 7th. Some of the advantages of the robot over standard laparoscopy are increased dexterity, improved visualization with high-resolution 3D image, tremor filtration, image fusion, and real-time integrated indocyanine green. However, it should be noticed that, despite the similar setting, robotic approach can be considered more similar to the traditional open surgery than to laparoscopy. In fact, the immersive experience offered by the robotic platform and the agility in the manipulation of tissues recall the environment of standard open technique. Moreover, the possibility of using a double console allows a higher level of interaction between the surgeon and the assistant for teaching purposes. It has been proposed that these features may allow a shorter learning curve as compared to standard laparoscopy [18]. Recently, Efanov and colleagues reported that the skills to perform difficult procedures with the robot are acquired faster compared to LLR, without differences in the incidence of postoperative complications [19].

Our experience shows statistically significant decrease of estimated blood loss, operative time, and low grade postoperative morbidities that can be interpreted as a crucial improvement to ensure patients' safety. The low incidence of high grade postoperative complication, as well as the low rate of readmissions, clearly demonstrates the ability of the robotic approach to minimize surgical impact. This turned to be even more important in the context of cirrhotic patients, which is evident in our cohort where 16.7% were Child B patients. In this setting of reduced liver function and functional reserve, we can benefit from gentle manipulation on the liver, respect of the venous shunts, and limited surgical trauma [20, 21]. Moreover, the results were confirmed also among the HCC subgroup (17 patients in the first group and 18 patients in the second group, p=0.79) and a safe resection was granted achieving always a negative margin in both populations (mean 10.4 cm and 17.1 cm, median 10 cm and 11 cm, respectively, p=0.22).

Indication to surgery and type of resection did not differ significantly between the two periods, and the different rate of major hepatectomies is not effect of patient selection. Moreover, after stratifying patients according to the score proposed by Wakabayashi and colleagues, we found that the two populations did not differ in terms of difficulty of the procedures. That means that the improvement observed is actually due to better surgical performances. Moreover, the incidence of intermediate level of difficulty prevailed in a statistically significant fashion over the study period. It may seem surprising, but it should be kept in mind that even if it represents the beginning of the robotic activity, the surgeon performing the procedures was already skilled in HPB and minimally invasive surgery [14]. The cultural background is crucial to develop new techniques, and our results mirror the experience of our center in the management of HPB surgery and postoperative care. We may hypothesize that the learning curve of residents and fellows should be focused on the correct use of the robotic platform, starting with easier procedures to get more confident with the console and the cart. Conversely, fully trained surgeons, in high volume referral centers, can approach intermediate level procedures along with some higher level ones. In the latter scenario, we showed that significant improvements in short-term surgical outcomes can be achieved after 30 cases. The development of robotic surgery for complex operations should be encouraged, but high quality standards and an ethics-driven surgical growth must be guaranteed [21].

## 5. Conclusions

Robotic approach proved to be safe and effective for the surgical treatment of liver diseases. In our experience, when analyzing the effects of a “mid-term activity,” a statistically significant improvement in both intra- and postoperative outcomes can be noticed. However, more studies are needed to confirm such results outside a HPB referral center. This is crucial, in particular to develop formal credentialing protocols for both junior and senior surgeons.

## Figures and Tables

**Table 1:** Patients characteristics and type of resection.

	First period (30)	Second period (30)	p-value
Age (years, mean)	57 (±14.1)	59 (±13.7)	0.65

Male/Female	21/9	20/10	0.78

Child A	28 (93.3%)	27 (90%)	0.64

Child B	2 (6.7%)	3 (10%)	0.64

Liver Cirrhosis	15 (50%)	17 (56.7%)	0.70

Right hepatectomy	2	0	0.15

Left hepatectomy	1	0	0.32

Bisegmentectomies (left lateral sectionectomies)	6 (5)	2 (0)	0.07 *(0.01)∗*

Segmentectomies	11	11	1

Wedge resections	10	14	0.3

Cystopericystectomies	1	3	0.3

**Table 2:** Perioperative data, histological findings, and difficulty index.

	First period (30)	Second period (30)	p-value
Conversion rate	0	1	0.32

Op. time (min, mean)	377.16	258.6	*<0.001∗*

Blood loss (ml, mean)	484.3	337.3	*0.01∗*

n. of nodules (mean)	1.27	1.23	0.77

Size (mm, mean)	34.6	28.4	0.16

Hosp. Stay (days, mean)	4.9 (2-13)	4.9 (2-12)	n.a.

Clavien I-II	19	6	*<0.001∗*

Clavien III-IV	2	1	0.56

Readmission	0	1	0.33

Mortality (30 d)	0	0	n.a.

HCC	17	18	0.79

CHC	0	1	0.32

Metastasis	5	6	0.74

Adenoma	4	2	0.40

Hydatic cyst	2	3	0.65

Low difficulty score	8 (26.7%)	10 (33.3%)	0.58

Intermediate difficulty score	17 (56.7%)	16 (53.3%)	0.79

High difficulty score	5 (16.7%)	4 (13.3%)	0.72

Modal score	5	5	

Median score	5	4.5	

Mean score	4.97	4.67	

Standard deviation	2.04	1.86	

Low vs Intermediate	*p= 0.03∗*	p= 0.13	

Low vs. High	p=0.39	*p=0.03∗*	

Intermediate vs. Low	*p=0.001∗*	*p<0.001∗*	

**Table 3:** Difficulty index variations.

Patient	Score	Difficulty	Patient	Score	Difficulty
1	7	high	31	6	intermediate

2	3	low	32	8	high

3	6	intermediate	33	1	low

4	6	intermediate	34	3	low

5	4	low	35	5	intermediate

6	9	high	36	5	intermediate

7	2	low	37	6	intermediate

8	4	low	38	5	intermediate

9	3	low	39	6	intermediate

10	5	intermediate	40	3	low

11	4	intermediate	41	8	high

12	7	high	42	2	low

13	5	intermediate	43	3	low

14	5	intermediate	44	5	intermediate

15	5	intermediate	45	3	low

16	10	high	46	3	low

17	4	intermediate	47	5	intermediate

18	1	low	48	4	intermediate

19	8	high	49	5	intermediate

20	6	intermediate	50	4	intermediate

21	4	intermediate	51	3	low

22	5	intermediate	52	3	low

23	5	intermediate	53	7	high

24	3	low	54	4	intermediate

25	5	intermediate	55	5	intermediate

26	6	intermediate	56	6	intermediate

27	6	intermediate	57	4	intermediate

28	5	intermediate	58	3	low

29	5	intermediate	59	9	high

30	1	low	60	6	intermediate

## Data Availability

The data used to support the findings of this study are included within the article.
